# Adding a Residential Dimension to the Scottish Population Spine – CHI-UPRN Residential Linkage (CURL).

**DOI:** 10.23889/ijpds.v7i3.1946

**Published:** 2022-08-25

**Authors:** David Clark, Chris Dibben

**Affiliations:** 1 Manitoba Centre for Healthy Policy; 2 Manitoba Metis Federation

## Objectives

In Spring 2020, Public Health Scotland (PHS) were tasked with seeding the NHS Scotland Community Health Index (CHI) with a Unique Property Reference Number (UPRN). This was to allow understanding of the impact on people residing with someone infected with COVID-19, but utility goes much further than this.

## Approach

Address fields were extracted from CHI records (August 2020) of persons still alive in Scotland or who died since January 2020.

Some pre-formatting was carried out on unique address strings that were then processed at DataHub, a service, provided by the Improvement Service, helping public sector organisations secure access to a data matching service.

Files were returned with a best matching UPRN attached, and a category indicating the quality of the match.

To enhance the seeding of care homes addresses, unmatched CHI records were manually searched, and UPRN added where expert knowledge determined it was a care home address.

## Results

There were 3,208,951 unique address strings processed, relating to 5,828,951 CHI registrations, including people deceased since January 2020. In total, 5,207,389 (89.3%) people had a UPRN returned with match categories EXCELLENT (78.8%), GOOD (8.3%), or FAIR (2.2%).

Only 60% of the 40,196 current CHI records that were pre-indicated with an institutional care home flag could be automatically matched to a UPRN. Following the manual expert review, 42,334 people in adult care homes could be identified with a specific UPRN.

A random sample of 1,724 address pairs, stratified by match category and urban-rural category, was selected for quality assurance review by an independent checker. Results have still to be verified, but precision is estimated to be more than 95% of matches to the correct UPRN.

## Conclusion

The creation of the CHI-UPRN Residential Linkage (CURL) is a major development to CHI as a data linkage tool in Scotland. CHI has long been used as a population spine for patient-level data linkage in research projects but introducing a property-level dimension through CURL provides new opportunities for researchers.

